# Predictors of Transitions From GADA as the Initial Autoantibody to Multiple Autoantibodies of Type 1 Diabetes in Children at Risk by a Dynamic Prediction Model

**DOI:** 10.1155/pedi/8845330

**Published:** 2025-09-16

**Authors:** Lu You, Falastin Salami, Roy Tamura, Carina Törn, Kendra Vehik, William A. Hagopian, Marian J. Rewers, Richard A. McIndoe, Jorma Toppari, Anette-G. Ziegler, Beena Akolkar, Jeffrey P. Krischer, Åke Lernmark

**Affiliations:** ^1^Health Informatics Institute, Morsani College of Medicine, University of South Florida, Tampa, Florida, USA; ^2^Department of Clinical Sciences, Lund University/Clinical Research Centre, Skåne University Hospital, Malmö, Sweden; ^3^Department of Pediatrics, Indiana University School of Medicine, Indianapolis, Indiana, USA; ^4^Barbara Davis Center for Childhood Diabetes, University of Colorado, Aurora, Colorado, USA; ^5^Center for Biotechnology and Genomic Medicine, Medical College of Georgia, Augusta University, Augusta, Georgia, USA; ^6^Department of Paediatrics, Turku University Hospital, Turku, Finland; ^7^Institute of Biomedicine, Research Centre for Integrated Physiology and Pharmacology and Centre for Population Health Research and InFlames Flagship Research Centre, University of Turku, Turku, Finland; ^8^Institute of Diabetes Research, Helmholtz Munich, German Research Center for Environmental Health, Munich, Germany; ^9^Forschergruppe Diabetes at Klinikum rechts der Isar, School of Medicine, Technical University Munich, Munich, Germany; ^10^Forschergruppe Diabetes e.V. at Helmholtz Munich, German Research Center for Environmental Health, Munich, Germany; ^11^Diabetes Branch, National Institute of Diabetes and Digestive and Kidney Diseases, Bethesda, Maryland, USA

**Keywords:** children, dynamic statistical modeling, GADA, risk prediction, type 1 diabetes

## Abstract

**Objective:** To design a dynamic prediction model for estimating the time of progression from a single glutamic acid decarboxylase autoantibody (GADA) to multiple islet autoantibodies and type 1 diabetes in children, exploring different longitudinally measured risk variables.

**Research Design and Methods:** GADA-positive children (*n* = 379) participating in The Environmental Determinants of Diabetes in the Young (TEDDY) study were followed for the appearance of additional autoantibodies against either insulin autoantibody (IAA), insulinoma-like 2 autoantibody (IA-2A), or zinc transporter 8 antibody (ZnT8A) and type 1 diabetes. A dynamic prediction model was designed, including trajectories of longitudinal risk variables, autoantibody titers, and metabolic variables (C-peptide, glucose, and HbA1c) together with time-invariant variables (gender, age at GADA positivity, and high-risk HLA genotypes).

**Results:** Transition risk from GADA to multiple autoantibodies was increased by lower age (*p* < 0.001) and by increased GADA titers during follow-up (*p* < 0.001), and was less likely in children with HLA DQ2/X but not DQ2/8 (*p*=0.004). The transition risk from multiple autoantibodies without IA-2A to IA-2A positivity was associated with increased levels of 2 h glucose following oral glucose tolerance test (OGTT) (*p* < 0.001) and increased ZnT8A titers (*p* < 0.001). Increasing HbA1c (*p* < 0.001) and GADA titers (*p* < 0.001) were associated with an increased risk of transition from GADA only to type 1 diabetes; while increasing HbA1c (*p* < 0.001) was associated with the transition from multiple autoantibodies to type 1 diabetes. Risk of transition from multiple autoantibodies, including IA-2A to type 1 diabetes was also associated with 2 h glucose level (*p* < 0.001).

**Conclusion:** The dynamic prediction model presented an individual time-specific risk of transition from a single GADA to multiple autoantibodies and type 1 diabetes.

## 1. Introduction

The global incidence of type 1 diabetes is rising among children, with approximately 90% of the cases present in the general population (GP) who do not have a family history of the disease [1, 2]. National screening has been urgently proposed, planned, or conducted in many countries in order to identify children at high risk for type 1 diabetes, predict different disease stages, and navigate to preventive trials. The complex pathogenesis and the variation in the progression among children make it difficult to predict the transition across the three presymptomatic (stages 1 and 2) and symptomatic (stage 3) type 1 diabetes stages [3]. Comprehensive and personalized prevention programs are urgently needed to inhibit or slow down the rising incidence of type 1 diabetes. Prediction models are also needed to tailor prevention programs directed at individualized transitioning across the different type 1 diabetes stages.

Type 1 diabetes is commonly preceded by seroconversion to one or multiple islet autoantibodies, including insulin autoantibodies (IAAs), glutamic acid decarboxylase autoantibodies (GADAs), insulinoma-like 2 autoantibodies (IA-2As), and zinc transporter 8 autoantibodies (ZnT8As). Multiplicity of these islet autoantibodies, along with a higher titer for each, raises the risk of developing clinical type 1 diabetes [1]. The two peaks of type 1 diabetes incidence in childhood occur in children between the ages of 4 and 7 years and in those between 10 and 14 years of age [4–6]. The most common first-appearing autoantibodies during these two age peaks are IAA (peaking in the first 2 years of life) and GADA (peaking between 4 and 6 years). GADA first is more common in older children than IAA [3, 7]. HbA1c is a known predictive biomarker that also rises along with autoantibody titers prior to the clinical onset of type 1 diabetes. We have previously reported that a certain increase in HbA1c within the normal reference range increased the risk for type 1 diabetes in children [8, 9]. The Environmental Determinants of Diabetes in the Young (TEDDY) study conducted in the United States, Sweden, Finland, and Germany screened approximately 8000 newborns for high-risk HLA-DQ-DR genotypes and followed them until 15 years of age or the onset of type 1 diabetes. TEDDY aimed to investigate genetic and environmental factors leading to autoimmunity as the primary endpoint and type 1 diabetes as the secondary endpoint [10]. The aim of this study is to present a statistical dynamic prediction model that predicts the time risk of developing multiple islet autoantibodies or the clinical onset of type 1 diabetes in children participating in the TEDDY study with GADA as the first appearing autoantibody.

## 2. Materials and Methods

### 2.1. Participants

TEDDY is a prospective observational cohort study conducted in the United States, Germany, Sweden, and Finland. The study was designed to investigate different factors, both environmental and genetic, associated with seroconversion and type 1 diabetes in children, as previously described [11]. TEDDY screened newborns for high-risk DQ/DR HLA genes at six clinical centers in Europe (Finland, Germany, and Sweden) and the United States between 2004 and 2010 and followed 8676 eligible children (89% without first-degree relative [FDR] with type 1 diabetes) until 15 years of age or the onset of type 1 diabetes [10]. This study included trajectories from all 379 children who became positive for GADA during the study follow-up period until June 30, 2023. Demographics and baseline characteristics of these 379 children are presented in Table 1. In the TEDDY study, written informed consent was obtained from a parent or primary caretaker for all participants, separately for genetic screening and for participation in the prospective follow-up. This study was approved by local Institutional Review Boards at participating sites and monitored by an External Advisory Board formed by the National Institutes of Health (NIH). Approvals included the Colorado Multiple Institutional Review Board (04-0361); Georgia's Medical College of Georgia Human Assurance Committee (2004–2010), Georgia Health Sciences University Human Assurance Committee (2011–2012), Georgia Regents University IRB (2013–2016), and Augusta University IRB (2017–present, HAC 0405380); University of Florida Health Center IRB (IRB201600277); Washington State IRB (2004–2012), Western IRB (2013–2019), and WCG IRB (2020–present, 20130211). European approvals came from Finland's Ethics Committee of the Hospital District of Southwest Finland (Dnro168/2004), Germany's Bayerischen Landesarztekammer Ethics Committee (04089), and Sweden's Regional Ethics Board in Lund (2004–2012), Lund University Committee for Continuing Ethical Review (2013–2021), and Swedish Ethical Review Authority (2022–present, 217/2004).

To understand the progression from GADA positivity to type 1 diabetes and two defined intermediate states; the transition from GADA as the single autoantibody to multiple autoantibodies without IA-2A or with IA-2A, a list of different variables (Table 2) was proposed to examine the correlation between these variables and the transition to the different islet autoantibody states or type 1 diabetes. IA-2A positivity is associated with a more rapid progression to stage 3 type 1 diabetes in children [12, 13]. This is why the risk of transitions was examined with or without IA-2A in this study.

### 2.2. HLA Genotyping

Children from the GP were included in the TEDDY study if they had one of the following high risk HLA genotypes: DRB1*⁣*^*∗*^04-DQA1*⁣*^*∗*^03-DQB1*⁣*^*∗*^03:02/DRB1*⁣*^*∗*^03-DQA1*⁣*^*∗*^05-DQB1*⁣*^*∗*^02:01 (DR3/4 or DQ2/8), DRB1*⁣*^*∗*^04-DQA1*⁣*^*∗*^03-DQB1*⁣*^*∗*^03:02/DRB1*⁣*^*∗*^04-DQA1*⁣*^*∗*^03-DQB1*⁣*^*∗*^03:02 (DR4/4 or DQ8/8), DRB1*⁣*^*∗*^04-DQA1*⁣*^*∗*^03-DQB1*⁣*^*∗*^03:02/DRB1*⁣*^*∗*^08-DQA1*⁣*^*∗*^04-DQB1*⁣*^*∗*^04:02 (DR4/8 or DQ8/X), and DRB1*⁣*^*∗*^03-DQA1*⁣*^*∗*^05-DQB1*⁣*^*∗*^02:01/DRB1*⁣*^*∗*^03-DQA1*⁣*^*∗*^05-DQB1*⁣*^*∗*^02:01 (DR3/3 or DQ2/2). Children with a FDR with type 1 diabetes could also have one of the following inclusion high risk genotypes: DRB1*⁣*^*∗*^04-DQA1*⁣*^*∗*^03-DQB1*⁣*^*∗*^03:02/DRB1*⁣*^*∗*^04-DQA1*⁣*^*∗*^03-DQB1*⁣*^*∗*^02:02 (DR4/4b or DQ8/X), DRB1*⁣*^*∗*^04-DQA1*⁣*^*∗*^03-DQB1*⁣*^*∗*^03:02/DRB1*⁣*^*∗*^01- DQA1*⁣*^*∗*^01-DQB1*⁣*^*∗*^05:01 (DR4/1 or DQ8/X), DRB1*⁣*^*∗*^04-DQA1*⁣*^*∗*^03-DQB1*⁣*^*∗*^03:02/DRB1*⁣*^*∗*^13-DQA1*⁣*^*∗*^01-DQB1*⁣*^*∗*^06:04 (DR4/13 or DQ8/X), DRB1*⁣*^*∗*^04-DQA1*⁣*^*∗*^03-DQB1*⁣*^*∗*^03:02/DRB1*⁣*^*∗*^09-DQA1*⁣*^*∗*^03-DQB1*⁣*^*∗*^03:03 (DR4/9 or DQ8/X), and DRB1*⁣*^*∗*^03-DQA1*⁣*^*∗*^05-DQB1*⁣*^*∗*^02:01/DRB1*⁣*^*∗*^09-DQA1*⁣*^*∗*^03-DQB1*⁣*^*∗*^03:03 (DR3/9 or DQ2/X) [14, 15]. The HLA screening was performed by each of the six clinical centers using various genotyping methods, as previously described [10], primarily from cord blood samples at birth. Additionally, some heel stick capillary samples for HLA screening were obtained up to the age of 4 months. As presented in Table 1, the children in this study were divided into three groups: (1) those having the greatest HLA genotype risk HLA DQ2/8, (2) one of the two highest risk haplotypes DQ8/X, or (3) DQ2/X.

### 2.3. Detection of Islet Autoantibodies

Islet autoantibodies were measured every 3 months until the age of 4 years, and measurements continued at the same frequency after seroconversion, or biannually if the child tested negative for islet autoantibodies (IAA, GADA, and IA-2A). Persistent autoimmunity was defined as the presence of any specific islet autoantibody on two consecutive visits and confirmed by two different core laboratories in TEDDY with high concordance. The four different types of islet autoantibodies were analyzed by different radiobinding assays (RBAs) with high sensitivity and specificity, detecting and quantifying the autoantibody titers as described previously [16–18]. The median baseline *z*-score titers for GADA, IAA, IA-2A, and ZnT8A are presented in Table 1.

### 2.4. Glycated Hemoglobin (HbA1c) Test

Once a TEDDY child tests positive for at least one islet autoantibody, a blood sample for HbA1c is drawn at the next clinical visit and at each subsequent clinical visit. The HbA1c samples were analyzed by ion-exchange HPLC methods (Tosoh G8 or Bio-Rad D-100), according to the National Glycohemoglobin Standardization Program (NGSP) laboratory network at the Diabetes Diagnostic Laboratory (DDL), University of Missouri, Columbia, as previously described [19, 20].

### 2.5. Oral Glucose Tolerance Test (OGTT)

OGTTs were performed biannually on children ≥3 years of age with two or more islet autoantibodies using either a 6-time-point OGTT (−10, 0, 30, 60, 90, and 120 min) or a two-time-point OGTT (0 and 120 min). The OGTT samples allowed for the analysis of fasting glucose, fasting C-peptide, and 120 min glucose and C-peptide at the TEDDY core laboratory, The Northwest Lipid Metabolism and Diabetes Research Laboratories, University of Washington, Seattle, as described elsewhere [21, 22] and University of Florida, Health Pathology Laboratories (UFHPL) Endocrine laboratory, Gainesville.

### 2.6. Statistical Methods and Data

A list of variables included in the dynamic model is presented in Table 2. OGTT measures and autoantibody titers were log(*x* + 1) transformed. The longitudinal variables were modeled by mixed effects models with splines. The population mean of the trajectories was captured by the fixed effects terms, and the inter-relation between longitudinal variables and their trajectories was captured by the random effects terms. We used polynomial splines to describe the trajectories of metabolic variables to reflect the smoothness of trajectories, while piecewise linear splines were used to describe the trajectories of autoantibody titers to capture the abrupt shifts. Several variables were log-transformed to facilitate a better fit to the mixed effects model.

**Figure 1:**
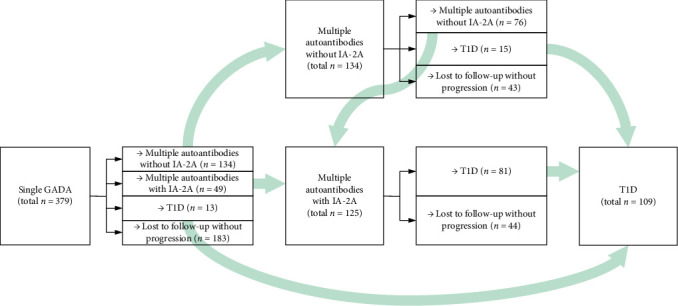
The diagram presents the number of children in the study group transitioning from GADA as a single islet autoantibody to multiple islet autoantibodies or type 1 diabetes and from multiple islet autoantibodies to type 1 diabetes.

The development of autoantibodies and type 1 diabetes was modeled by a multistate model with four states, single GADA positive (abbreviated as “single GADA”), multiple islet autoantibodies without IA-2A (abbreviated as “multiple without IA-2A”), multiple islet autoantibodies with IA-2A (abbreviated as “multiple with IA-2A”), and type 1 diabetes. Transitions between the four states can be described by the diagram presented in Figure 1. For each transition, we proposed a list of variables to investigate their relationship. A full list of proposed variables is presented in Table 3. A proportional hazards model was used to assess the relationship between variables and risk of transitions, where the linear increase in variables corresponded to a proportional change in the risk of transitions. The risk of transitions was measured by a hazard function as a function of the time since these children became positive for GADA. We additionally applied the least absolute shrinkage and selection operator (LASSO) regularization technique to determine variables with little associations to the risk of transitions and shrank them toward zero [23]. A coefficient of zero indicated that the variable was excluded by the LASSO process, as it did not significantly contribute to predicting that specific transition.

A joint model of longitudinal data and multistate data was used to simultaneously estimate the parameters in the longitudinal and multistate models [24]. The joint model was then used to make dynamic predictions. For example, suppose we have collected several years of data following GADA positivity from a new participant, the future trajectories of longitudinal variables can then be inferred from the mixed effects model. Combining the future trajectories with the multistate model, we can obtain the estimated probability of occupying each state following the current time. The prediction is dynamic as it can be updated as new data become available.

## 3. Results

### 3.1. Number of Children Transitioning From Single GADA to the Next State

The observed number of children in the study group transitioning from GADA as a single autoantibody to different autoantibody statuses (multiple autoantibodies without IA-2A and multiple autoantibodies with IA-2A) and type 1 diabetes (single autoantibody to type 1 diabetes, multiple autoantibodies without IA-2A to type 1 diabetes, and multiple autoantibodies with IA-2A to type 1 diabetes) is presented in the DAG diagram in Figure 1.

## 4. Multistate Model Results

### 4.1. Single GADA to Multiple Islet Autoantibodies and Type 1 Diabetes

The risk of transition from a single GADA to multiple islet autoantibodies with or without IA-2A was significantly associated with higher GADA titers (*p* < 0.001; *p* < 0.001, respectively) and younger age (*p* < 0.001; *p*=0.009, respectively) during the longitudinal follow-up. Having the genotype DQ2/X but not DQ2/8 had a negative effect (lower risk) on the risk of transition from a single GADA to multiple autoantibodies without IA-2A (*p*=0.004). The transition risk from a single GADA to type 1 diabetes was associated with higher GADA titers (*p* < 0.001) and increased HbA1c levels (*p* < 0.001). The other proposed risk variables did not show any correlation with these transitions (Table 3).

### 4.2. Multiple Islet Autoantibodies Without IA-2A to Multiple With IA-2A and Type 1 Diabetes

The development of IA-2A following persistent multiple islet autoantibodies was associated with increased OGTT 2 h p-glucose levels (*p* < 0.001) and increased ZnT8A (*p* < 0.001) and IAA titers (*p*=0.017). The risk of transition was lower in children carrying the genotype HLA DQ2/X compared to children with HLA DQ2/8 (*p*=0.007). Transitioning from multiple islet autoantibodies without IA-2A to type 1 diabetes was associated with increased HbA1c levels during follow-up. No correlation was found between other proposed variables and these transitions (Table 3).

### 4.3. Multiple Islet Autoantibodies With IA-2A to Type 1 Diabetes

The development of type 1 diabetes following multiple islet autoantibodies with IA-2A was associated with being a FDR (*p* < 0.001), increased HbA1c levels (*p* < 0.001), and increased 2 h glucose levels (*p* < 0.001) during the longitudinal follow-up. No other risk variables were correlated with this transition (Table 3).

### 4.4. Change of Longitudinal Proposed Risk Factors

**Figure 2:**
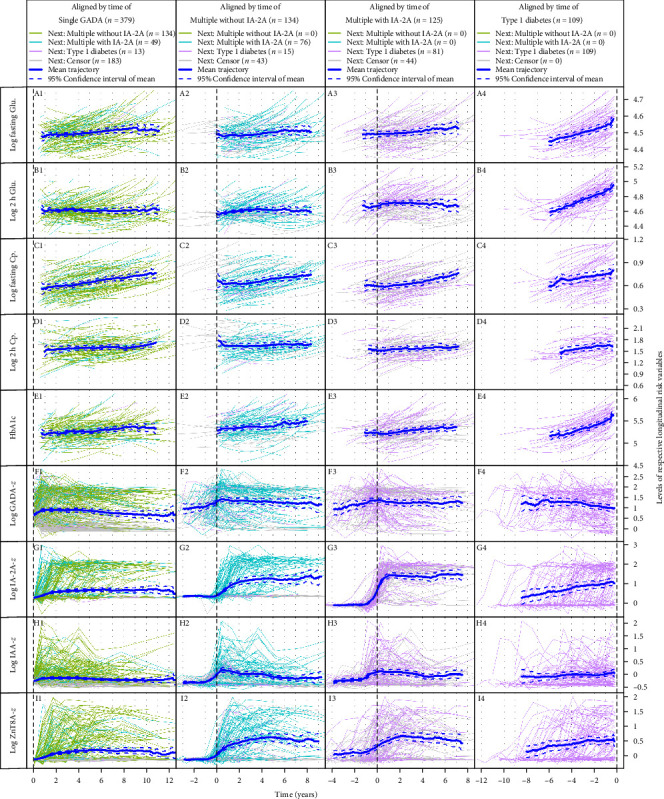
Change of longitudinal risk factors. Each line presents trajectories for one child, the blue solid bolded line presents the population mean trajectory and the blue dashed lines represent the 95% CI for the population mean trajectory. Aligned by time of the different transition states presented in the columns. Abbreviations: Cp, C-peptide; Glu, glucose.

Trajectories of different proposed longitudinal risk factors aligned by time of transition to different states are presented for each included child, with the mean population trajectory with 95% confidence interval (CI) in Figure 2.

As visualized in the diagram in Figure 2, fasting glucose (Panel A4), 2 h glucose (Panel B4), and HbA1c (Panel E4) showed a clear upward trend prior to type 1 diabetes. IA-2A (Panel G4) and ZnT8A (Panel I4) levels also showed increasing trends prior to type 1 diabetes, while the fasting C-peptide levels increased steadily over time in all groups. The GADA titers decreased slightly after the transition to the second or multiple islet autoantibodies without IA-2A and prior to type 1 diabetes onset.

### 4.5. Dynamic Prediction Model

A dynamic prediction model, based on the joint models, was employed to make predictions using the study data. We now illustrate the dynamic prediction model for a male child with HLA DQ2/8 who developed GADA at the age of 2.3 years without a family history of type 1 diabetes in FDRs.

**Figure 3:**
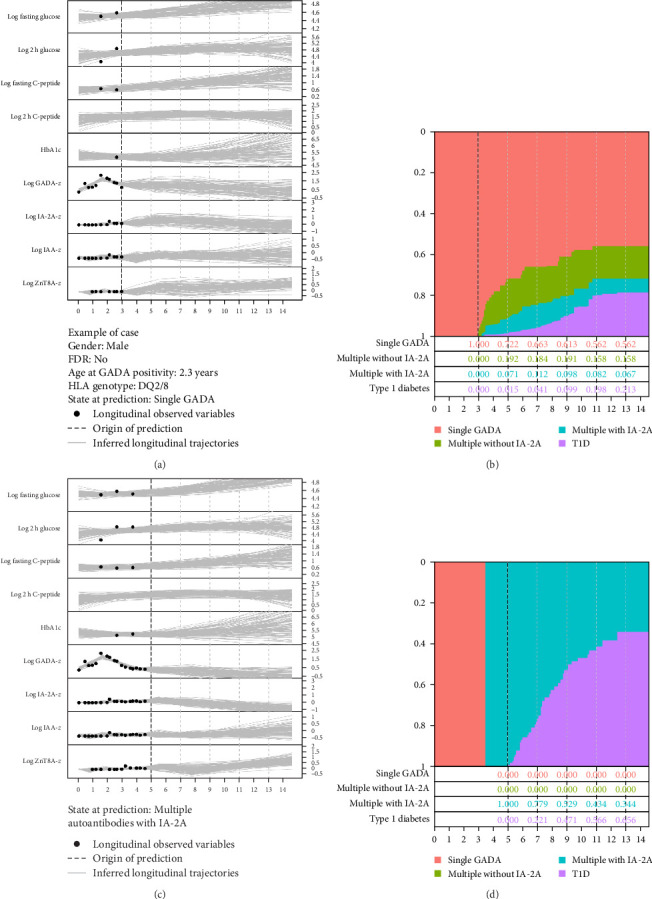
Dynamic prediction of islet autoantibody status and type 1 diabetes from a TEDDY boy who tested positive for GADA at 2.3 years of age. (A) Data input of all observed longitudinal variables 3 years after GADA positivity, resulting in inferred longitudinal trajectories. (B) Dynamic prediction model predicting the different states of islet autoantibodies and type 1 diabetes 3 years from GADA positivity. (C) Data input of all observed longitudinal variables 5 years after GADA positivity, resulting in inferred longitudinal trajectories. (D) Dynamic prediction model predicting the risk of transition from multiple islet autoantibodies with IA-2A to type 1 diabetes.

We first assume that the participant was followed for 3 years after GADA positivity and was still single GADA positive. 3 years of longitudinal data (Table 2) following GADA positivity and the history of autoantibody status were used as data inputs. The past and future longitudinal trajectories are inferred from the observed data (Figure 3A). The dynamic prediction model presented time-dependent probabilities of occupying a certain islet autoantibody state or progression to type 1 diabetes after GADA positivity, as illustrated in Figure 3B. Next, we assumed that 2 years of additional longitudinal data were collected, and we observed that the participant had multiple autoantibodies with IA-2A positive between 3 and 4 years after GADA positivity. The inferred longitudinal trajectories and updated probabilities of state occupation and type 1 diabetes are presented in Figure 3C, D. The 2 years of additional data suggested that the individual was at higher risk of type 1 diabetes. The dynamic prediction model is available through the web-based interface at https://luyouepiusf.shinyapps.io/rgadadynamicprediction_shinyio/.

## 5. Discussion

The time prediction of the development of multiple islet autoantibodies (stage 2 type 1 diabetes) or onset of clinical type 1 diabetes is still very hard to achieve [25]. The prediction of the time to stage 2 type 1 diabetes is important for early interventions to delay or stop the ongoing autoimmune destruction of the islet beta cells. Thus, prediction of time to the disease is important to prevent ketoacidosis and to control hyperglycemia for better individual treatment and individual monitoring to avoid severe long-term diabetic complications. We hereby present a dynamic prediction model to predict time to multiple islet autoantibodies or type 1 diabetes onset, following GADA as the single islet autoantibody in children.

The TEDDY study's multinational cohort, which involves a majority of participants (89%) without an FDR with type 1 diabetes, reflects the GP [26]. The study's detailed and regular longitudinal follow-up of children at risk enhances the robustness of the data. Consequently, the dynamic prediction model based on data from 379 GADA-positive TEDDY children is highly generalizable and could be effectively utilized in other longitudinal programs to predict the time of disease progression. However, this dynamic prediction model is specifically tailored for TEDDY children who are carefully selected based on high-risk HLA genotypes. If modified to exclude HLA, the model could potentially be adapted for population-wide screening programs, which typically do not incorporate HLA genotyping but rely solely on autoantibody status and titers. However, applying the model to populations with different HLA risk or without HLA information may shift the predictive weights of other variables. Also, while clinicians can input similar longitudinal data points (e.g., OGTT and autoantibody titers) for individual patients into the dynamic prediction model, access to a rich data history comparable to the TEDDY study is required.

One limitation of the dynamic prediction model presented in this study is that it is only applicable to children who develop GADA as their first islet autoantibody. Therefore, children who develop IAA, IA-2A, or ZnT8A as their first islet autoantibody will require distinct dynamic prediction models designed for each of these specific islet autoantibodies. However, GADA is the most common first-appearing islet autoantibody in >4–5 years old children and adolescents preceding type 1 diabetes [27–29].

The transition risk from GADA as a single islet autoantibody to multiple islet autoantibodies, including or excluding IA-2A was associated with younger age and increased GADA titers over time. It is well known that the younger a child is when developing the first islet autoantibody, the higher the risk of rapidly developing multiple autoantibodies and progressing to type 1 diabetes diagnosis [7, 30]. This may be due to a vulnerable, still-developing immune system leading to a stronger autoimmune response and a more rapid progression to stage 2 and stage 3 type 1 diabetes [7, 31]. Increased GADA titers are a sign of increased autoimmune attack on the beta cells, leading to autoantibody spreading and the development of multiple islet autoantibodies [12]. Having HLA DQ2/8 conferring the highest HLA genotype risk was associated with the transition from a single GADA to multiple islet autoantibodies without IA-2A during follow-up. The transition risk from multiple islet autoantibodies to multiple islet autoantibodies, including IA-2A was associated with increased 2 h p-glucose (OGTT) and ZnT8A titers, mainly in children with HLA DQ2/8, indicating a progressive autoimmune response with impaired beta cell function.

Higher GADA levels during follow-up were associated with a higher risk of stage 3. However, previous reports in the literature have been inconsistent, with some studies supporting our findings and others contradicting them [31–33]. Conversely, the mean trajectory for the cohort showed a decrease in GADA titers over time prior to type 1 diabetes onset, as illustrated in Figure 2 (Panel F4). These inconsistencies in the literature may be due to variations in the age range and duration of follow-up, as our study spans from birth to 15 years of age. Our results indicate that GADA titers increase during the follow-up period in association with the onset of type 1 diabetes, but this rise appears to decline within the 2 years immediately preceding onset. In this study, a proportional hazards model is used to assess the relative risk of progression compared to the population average. This implies that individuals with higher GADA titers than the population average are more likely to progress more rapidly. While it may seem contradictory, that decreases in GADA do not necessarily suggest a lower progression rate to type 1 diabetes. This reflects distinctions in population-level trends and individual-level variation relative to the population, which are not mutually exclusive. The other variable associated with the transition risk from a single GADA to type 1 diabetes is increasing HbA1c levels during follow-up as reported previously in TEDDY [8, 9].

The trajectories for the different variables perceived as risk factors (Figure 2) showed an increase in IA-2A and ZnT8A titers (Panel G4 and I4), as well as glucose metabolism measures, like HbA1c (Panel E4), fasting glucose (Panel A4) and 2 h glucose (OGTT) (Panel B4) years prior to type 1 diabetes, reflecting a progressive autoimmune destruction of the pancreatic beta-cells leading to loss of beta cell function and an impaired insulin production [34, 35]. Trajectories of C-peptide demonstrated an increase in fasting C-peptide levels over time, which occurs as children grow and their energy demands rise in response to growth and increased BMI. One limitation of this analysis is the absence of OGTT data for children with a single islet autoantibody. In the TEDDY study, OGTT is only performed in children with multiple islet autoantibodies. Including OGTT data for children with GADA alone could have potentially enhanced the accuracy of the dynamic prediction model.

## 6. Conclusion

We are presenting a statistical model designed as a tool for the prediction of progression to stage 3 type 1 diabetes. This model estimates the probable time of developing multiple islet autoantibodies, stage 2 type 1 diabetes, or the onset of stage 3 type 1 diabetes. As the progression to the different stages of type 1 diabetes is highly heterogeneous among at-risk children, such a model can be effectively used in various surveillance programs, including national health screening programs, to predict the disease in children. It can also be utilized in different longitudinal studies to guide appropriate interventions or preventions to slow or prevent the autoimmune process leading to type 1 diabetes and severe clinical hyperglycemic long- and short-term complications.

## Figures and Tables

**Table 1:** Demographics and baseline characteristics of the 379 GADA-positive children.

Variable	Frequency (percent) or median (IQR)
Sex	
Female	174(45.9%)
Male	205(54.1%)
Country	
Finland	76(20.1%)
Germany	20(5.3%)
Sweden	143(37.7%)
United States	140(36.9%)
Age	5.1(2.4,9.0)
FDR	66(17.4%)
DQ group	
DQ2/8	185(48.8%)
DQ8/X (not DQ2/8)	112(29.6%)
DQ2/X (not DQ2/8)	82(21.6%)
Fasting glucose (mg/dL)	88(83,94)
2 h Glucose (mg/dL)	106(96,120)
Fasting C-peptide (ng/mL)	0.98(0.67,1.25)
2 h C-peptide (ng/mL)	3.77(2.75,5.00)
GADA-*z* titers	0.417(0.135,1.192)
IA-2A-*z* titers	−0.087(−0.087,−0.087)
IAA-*z* titers	−0.322(−0.322,−0.315)
ZnT8A-*z* titers	−0.105(−0.127,−0.089)

*Note: z*: statistical *z* score for islet autoantibody titers.

Abbreviation: FDR, first-degree relative.

**Table 2:** Proposed risk variables for the transition from GADA as a single islet autoantibody to multiple islet autoantibodies and type 1 diabetes.

Longitudinal variables
Metabolic variables Fasting glucose (fasting glu.) (log-transformed) 2 h glucose (2 h glu.) (log-transformed) Fasting C-peptide (fasting cp.) (log-transformed) 2 h C-peptide (2 h cp.) (log-transformed) HbA1c
Islet autoantibody titers GADA titers (GADA-*z*) (log-transformed) IA-2A titers (IA-2A-*z*) (log-transformed) IAA titers (IAA-*z*) (log-transformed) ZnT8A titers (ZnT8A-*z*) (log-transformed)
Nontime-varying variables
Sex
Age at confirmed GADA positive
FDR
HLA genotype DQ8/X (not including DQ2/8, DQ2/8 is the reference group) DQ2/X (not including DQ2/8, DQ2/8 is the reference group)

**Table 3:** Correlation between risk variables and the transition from GADA as the single islet autoantibody to multiple islet autoantibody states (with or without IA-2A) or type 1 diabetes, from multiple islet autoantibodies to IA-2A and from multiple islet autoantibodies (with or without IA-2A) to type 1 diabetes.

Transitions from one state to another	Variable	Coefficient	Std. err	*p*-Value
Single GADA --> multiple without IA-2A	**Log GADA-*z*** ^a^	**0.995**	**0.119**	**<0.001**
Single GADA --> multiple without IA-2A	FDR	0.126	0.221	0.570
Single GADA --> multiple without IA-2A	**Age**	**−0.124**	**0.0280**	**<0.001**
Single GADA --> multiple without IA-2A	DQ8/X	−0.0625	0.206	0.761
Single GADA --> multiple without IA-2A	DQ2/X	**−0.665**	**0.233**	**0.004**
Single GADA --> multiple with IA-2A	**Log GADA-*z***	**1.27**	**0.205**	**<0.001**
Single GADA --> multiple with IA-2A	FDR	0.306	0.343	0.373
Single GADA --> multiple with IA-2A	**Age**	**−0.121**	**0.0465**	**0.009**
Single GADA --> multiple with IA-2A	DQ8/X	0	0.345	—
Single GADA --> multiple with IA-2A	DQ2/X	−0.615	0.372	0.099
Single GADA --> type 1 diabetes	**HbA1c**	**3.89**	**0.995**	**<0.001**
Single GADA --> type 1 diabetes	**Log GADA-z**	**1.48**	**0.406**	**<0.001**
Single GADA --> type 1 diabetes	FDR	0.678	0.662	0.306
Single GADA --> type 1 diabetes	Age	−0.140	0.0978	0.154
Single GADA --> type 1 diabetes	DQ8/X	0	0.867	—
Single GADA --> type 1 diabetes	DQ2/X	1.08	0.677	0.109
Multiple without IA-2A --> multiple with IA-2A	Log fasting glucose	0	2.21	—
Multiple without IA-2A --> multiple with IA-2A	**Log 2 h glucose**	**3.48**	**0.877**	**<0.001**
Multiple without IA-2A --> multiple with IA-2A	Log fasting C-peptide	0	1.02	—
Multiple without IA-2A --> multiple with IA-2A	Log 2 h C-peptide	−1.14	0.603	0.058
Multiple without IA-2A --> multiple with IA-2A	HbA1c	0	0.597	—
Multiple without IA-2A --> multiple with IA-2A	Log GADA-*z*	0.156	0.185	0.399
Multiple without IA-2A --> multiple with IA-2A	**Log IAA-*z***	**0.616**	**0.258**	**0.017**
Multiple without IA-2A --> multiple with IA-2A	**Log ZnT8A-*z***	**1.28**	**0.230**	**<0.001**
Multiple without IA-2A --> multiple with IA-2A	FDR	0.216	0.302	0.476
Multiple without IA-2A --> multiple with IA-2A	Age	0	0.0488	—
Multiple without IA-2A --> multiple with IA-2A	DQ8/X	0.0556	0.269	0.836
Multiple without IA-2A --> multiple with IA-2A	**DQ2/X**	**−1.22**	**0.451**	**0.007**
Multiple without IA-2A --> type 1 diabetes	Log fasting glucose	8.66	5.67	0.127
Multiple without IA-2A --> type 1 diabetes	Log 2 h glucose	1.51	2.08	0.468
Multiple without IA-2A --> type 1 diabetes	Log fasting C-peptide	0	2.31	—
Multiple without IA-2A --> type 1 diabetes	Log 2 h C-peptide	−2.68	1.40	0.056
Multiple without IA-2A --> type 1 diabetes	**HbA1c**	**7.14**	**1.57**	**<0.001**
Multiple without IA-2A --> type 1 diabetes	Log GADA-*z*	0	0.402	—
Multiple without IA-2A --> type 1 diabetes	Log IAA-*z*	0.389	1.00	0.697
Multiple without IA-2A --> type 1 diabetes	Log ZnT8A-*z*	0.655	0.553	0.237
Multiple without IA-2A --> type 1 diabetes	FDR	−0.678	0.877	0.439
Multiple without IA-2A --> type 1 diabetes	Age	0	0.135	—
Multiple without IA-2A --> type 1 diabetes	DQ8/X	0	0.769	—
Multiple without IA-2A --> type 1 diabetes	DQ2/X	0.830	0.765	0.278
Multiple with IA-2A --> type 1 diabetes	Log fasting glucose	2.13	2.13	0.317
Multiple with IA-2A --> type 1 diabetes	**Log 2 h glucose**	**4.71**	**0.938**	**<0.001**
Multiple with IA-2A --> type 1 diabetes	Log fasting C-peptide	−0.00169	1.13	0.999
Multiple with IA-2A --> type 1 diabetes	Log 2 h C-peptide	0	0.698	—
Multiple with IA-2A --> type 1 diabetes	**HbA1c**	**2.01**	**0.507**	**<0.001**
Multiple with IA-2A --> type 1 diabetes	Log GADA-*z*	−0.224	0.174	0.198
Multiple with IA-2A --> type 1 diabetes	Log IA-2A-*z*	0	0.214	—
Multiple with IA-2A --> type 1 diabetes	Log IAA-*z*	0	0.306	—
Multiple with IA-2A --> type 1 diabetes	Log ZnT8A-*z*	−0.260	0.232	0.263
Multiple with IA-2A --> type 1 diabetes	**FDR**	**1.17**	**0.301**	**<0.001**
Multiple with IA-2A --> type 1 diabetes	Age	−0.0702	0.0668	0.293
Multiple with IA-2A --> type 1 diabetes	DQ8/X	0	0.296	—
Multiple with IA-2A --> type 1 diabetes	DQ2/X	0	0.381	—

*Note:* Coefficients for DQ8/X and DQ2/X represent the change in risk for participants having DQ8/X genotypes (but not DQ2/8) and having DQ2/X genotypes (but not DQ2/8) in reference to the DQ2/8 group. Coefficients of “0” and *p*-values of “—” indicate variables excluded by the LASSO regularization process, as they showed minimal evidence contributing to predict the specific transitions. Bold indicates the significant values.

^a^-*z*: *z*-score.

## Data Availability

Data from The Environmental Determinants of Diabetes in the Young (https://doi.org/10.58020/y3jk-x087) reported here will be made available for request at the NIDDK Central Repository (NIDDK-CR) website, Resources for Research (R4R), https://repository.niddk.nih.gov/. The dynamic prediction model is available through the web-based interface at https://luyouepiusf.shinyapps.io/rgadadynamicprediction_shinyio/.

## Supporting Information

Supporting Information Members of the TEDDY study group listed (one document).
